# The Human Gut Microbe *Bacteroides thetaiotaomicron* Suppresses Toxin Release from *Clostridium difficile* by Inhibiting Autolysis

**DOI:** 10.3390/antibiotics10020187

**Published:** 2021-02-15

**Authors:** Miad Elahi, Haruyuki Nakayama-Imaohji, Masahito Hashimoto, Ayano Tada, Hisashi Yamasaki, Tamiko Nagao, Tomomi Kuwahara

**Affiliations:** 1 Department of Microbiology, Faculty of Medicine, Kagawa University, 1750-1 Miki, Kagawa 761-0793, Japan; miadelahiabesh@gmail.com (M.E.); imaohji@med.kagawa-u.ac.jp (H.N.-I.); ayatada@med.kagawa-u.ac.jp (A.T.); 2 Department of Chemistry, Biotechnology, and Chemical Engineering, Kagoshima University, Kagoshima 890-8580, Japan; hassy@eng.kagoshima-u.ac.jp; 3 Division of Biology, Hyogo College of Medicine, Mukogawa, Nishinomiya 663-8501, Japan; hi-yamasaki@hyo-med.ac.jp; 4 Department of Science for Human Health Welfare Care Major, Shikoku University Junior College, Ohjin, Tokushima 771-1192, Japan; tnagao@shikoku-u.ac.jp

**Keywords:** Bacteroides thetaiotaomicron, Clostridium difficile, toxin, autolysis, cell wall

## Abstract

Disruption of the human gut microbiota by antibiotics can lead to *Clostridium difficile* (CD)-associated diarrhea. CD overgrowth and elevated CD toxins result in gut inflammation. Herein, we report that a gut symbiont, *Bacteroides thetaiotaomicron* (BT), suppressed CD toxin production. The suppressive components are present in BT culture supernatant and are both heat- and proteinase K-resistant. Transposon-based mutagenesis indicated that the polysaccharide metabolism of BT is involved in the inhibitory effect. Among the genes identified, we focus on the methylerythritol 4-phosphate pathway gene *gcpE*, which supplies the isoprenoid backbone to produce the undecaprenyl phosphate lipid carrier that transports oligosaccharides across the membrane. Polysaccharide fractions prepared from the BT culture suppressed CD toxin production in vitro; the inhibitory effect of polysaccharide fractions was reduced in the *gcpE* mutant (Δ*gcpE*). The inhibitory effect of BT-derived polysaccharide fraction was abrogated by lysozyme treatment, indicating that cellwall-associated glycans are attributable to the inhibitory effect. BT-derived polysaccharide fraction did not affect CD toxin gene expression or intracellular toxin levels. An autolysis assay showed that CD cell autolysis was suppressed by BT-derived polysaccharide fraction, but the effect was reduced with that of Δ*gcpE*. These results indicate that cell wall-associated glycans of BT suppress CD toxin release by inhibiting cell autolysis.

## 1. Introduction

*Clostridium difficile* (CD) is a Gram-positive, spore-forming, rod-shaped anaerobe. CD is a leading cause of nosocomial infectious diarrhea, especially in elderly patients at hospitals and nursing homes [1]. CD can progress to severe, even deadly, conditions such as pseudomembranous colitis, toxic megacolon, and colonic perforation [2]. Patients with CD-associated diarrhea (CDAD) excrete CD spores in the feces. CD spores are resistant to antimicrobial agents, contaminate environmental surfaces, and transmit CD among patients [3,4]. Strict contact precautions are required for CDAD patients to prevent nosocomial outbreaks of this pathogen. CDAD increases healthcare costs due to extended hospital stays of CDAD patients and infection countermeasures [5].

CDAD is caused by CD production of macromolecular toxins called toxin A (308 kDa) and toxin B (297 kDa) [6,7]. These toxins induce actin disaggregation by glucosylating host Rho GTPase [8], which compromises gut barrier function and results in gut epithelial cell death. Non-toxigenic CD is not pathogenic, rather it has been utilized tentatively to outcompete diarrheagenic CD [9], indicating that these toxins are crucial to the determination of CD pathogenicity.

Toxins A and B are encoded in a 19.6-kb pathogenicity locus (known as PaLoc), which is exclusively present in the chromosome of diarrheagenic CD [10]. This locus includes the genes for a transcriptional regulator (*tcdR* and *tcdC* for activator and suppressor, respectively) and a holin-like protein (*tcdE*) [11,12,13]. The *tcdE* gene product has been suggested to facilitate the extracellular release of CD toxins. [14]. Over the past few decades, the hypervirulent CD lineage NAP1/B1/027 has emerged and spread worldwide [15]. This high virulence CD lineage produces large amounts of toxins A and B and has an additional toxin (binary toxin) encoded in another genomic locus [16,17]. The high morbidity and mortality rate of the NAP1/B1/027 lineage, as well as its higher transmissibility, makes CDAD one of the major public health concerns worldwide [18,19].

The human gut harbors diverse and numerous microbes that deeply associate with host physiology. This ecosystem provides beneficial effects to the host such as energy extraction from otherwise indigestible dietary polysaccharides, gut immune system maturation, and colonization resistance to enteric pathogens. Antibiotic treatment compromises gut microbiota function and is recognized as the most important risk factor for CDAD [20]. The reduced colonization resistance caused by antibiotic therapy allows CD to overgrow, which increases the toxin level in the gut. Although the first line therapy for CDAD is antibiotic treatment with either vancomycin or metronidazole [21], recurrence is observed in nearly 20% of patients [22]. In addition, the relapse rate increases by 20% with each recurrent episode, and the cure rate with antibiotic therapy for these cases is only 25 to 35% [23,24]. Recently, fecal microbiota transplantation (FMT) has been used to treat CDAD cases refractory to antibiotic therapy [25]. The therapeutic efficacy of FMT has been reported at over 90% [26], indicating that restoration of the gut microbiota function is essential for remission from recurrent CDAD.

Besides its efficacy, there are concerns about FMTs such as the transmission of unknown pathogens to recipients and a lack of selection guidelines for donor feces. Most importantly, the underlying mechanism for the therapeutic efficacy of FMTs remains to be elucidated. Lawley et al. reported that bacteriotherapy using a simple mixture of six intestinal bacteria—*Staphylococcus warneri*, *Enterococcus hirae*, *Lactobacillus reuteri,* as well as three novel species of *Anaerostipes*, *Enterorhabdus,* and *Bacteroides*—resolved CD disease and transmission in a mouse model [27]. The results indicate that colonization resistance and/or anti-pathogenic potential of the normal gut microbiota against CD is attributable to a particular set of gut microbes.

*Bacteroides* is a Gram-negative, rod-shaped anaerobe, which is among the most predominant members of the gut microbiota. Cordonnier et al. have reported that *B. thetaiotaomicron* suppresses the Shiga-toxin production of enterohemorrhagic *Escherichia coli* by competing with vitamin B_12_ uptake [28]. It has also been reported that the related species *B. fragilis* prevents CD infection by facilitating the restoration of gut barrier function and microbiota composition [29]. Thus, *Bacteroides* plays a key role in maintaining the integrity of the human gut. However, the direct action of *Bacteroides* spp. against the pathogenic mechanisms of CD remains to be elucidated. In this study, we examined the effect of *Bacteroides* spp. on the toxigenicity of CD and found that *B. thetaiotaomicron* suppressed CD toxin production. Herein, we report how this gut symbiont affects the toxin production of CD.

## 2. Results

### 2.1. Effect of Bacteroides Species on CD Toxin Production

**Figure 1:**
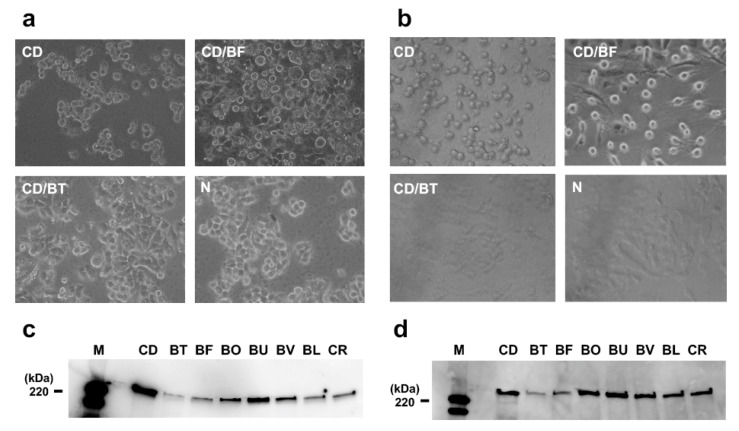
**Effect of *Bacteroides* species on *Clostridium difficile* (CD) toxin production.** Cytopathic effect by CD toxins A and B in the culture supernatants were evaluated by a cytotoxicity assay using (**a**) human colon cancer cell line HT-29 cells and (**b**) Vero cells. CD, CD ATCC9689; CD/BF, co-culture of CD ATCC9689 and *B. fragilis* YCH46; CD/BT, co-culture of CD ATCC9689 and *B. thetaiotaomicron* VPI-5482; and N, GAM broth only. Western blotting of the cell-free CD supernatants in the conditioned media with either (**c**) anti-Toxin A or (**d**) anti-Toxin B antibody. CD, CD ATCC9689 culture supernatant in GAM broth; conditioned medium containing 50% (*v*/*v*) culture supernatant of BT, *B. thetaiotaomicron* VPI-5482; BF, *B. fragilis* YCH46; BO, *B. ovatus* ATCC8483; BU, *B. uniformis* ATCC8492; BV, *B. vulgatus* ATCC8503; BL, *Bifodobacterium longum* JCM1217; and CR, *Clostridium ramosum* JCM1298. M, HPR-labelled protein size marker.

We first examined the effect of *Bacteroides* species on CD toxin production. CD ATCC9689 was co-cultured with the selected *Bacteroides* species. Supernatants of the 24-h co-cultures were filter-sterilized and equally mixed with Dulbecco’s Modified Eagle’s Medium (DMEM) containing 10% fetal bovine serum (FBS). Cytopathic effects by toxin A and B in these cultures were evaluated in the human colon cancer cell line HT29 (Figure 1a) and Vero cells (Figure 1b), respectively. After the cells were cultured to semi-confluent, DMEM was replaced with a DMEM containing 50% of the filter-sterilized supernatant prepared from co-culture of CD and *Bacteroides* species. The cells were further incubated for 24 h. These cells turned round following exposure to CD culture supernatant (CD in Figure 1). The cell rounding was suppressed (see CD/BT in Figure 1) when CD was co-cultured with *B. thetaiotaomicron* VPI-5482 (BT), and the cell morphology was similar to that of the Gifu Anaerobic Medium (GAM) control (N in Figure 1). Western blotting was performed with anti-CD toxin A and B antibody against CD culture supernatant from the conditioned medium containing the culture supernatant (50% *v*/*v*) of each selected human gut microbe (Figure 1c,d). The results showed that both toxin A and toxin B production was reduced in the conditioned medium prepared with BT culture supernatant. These data indicate that BT culture supernatant contains anti-toxigenic factors to CD. *B. fragilis* (BF) culture supernatant also reduced the toxin levels, but the effect was less than that of BT. Other *Bacteroides* species tested did not have an effect on CD toxin production. To determine whether the effect was strain-specific, the conditioned media were prepared with seven clinical isolates of BT. All the BT strains tested suppressed CD toxin A production (Figure S1).

### 2.2. Characterization of BT-Derived Inhibitory Factors on CD Toxin Production

**Figure 2:**
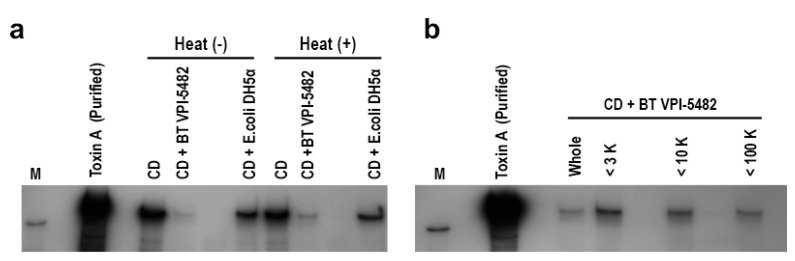
**Characterization of BT-derived inhibitory factors on CD toxin production.** (**a**) Heat stability was examined by heating the culture supernatant of BT or *Escherichia coli* DH5α at 100 °C for 10 min. After CD was cultured in either GAM broth or the conditioned media containing either heated- or unheated-culture supernatant, Toxin A level in the culture was compared by Western blotting with anti-Toxin A antibody. (**b**) Size fractionation of the culture supernatant of BT by Amicon^®^ ultrafiltration column. Filtration sizes are indicated above the lanes. “Whole” indicates the unfiltered sample. CD was cultured in the conditioned media prepared with either the filtered or unfiltered BT culture supernatant; Toxin A level in the culture was compared by Western blotting with anti-Toxin A antibody. M, HRP-labeled protein size marker (220 kDa).

We examined the heat stability and estimated the sizes of inhibitory factors on CD toxin production, which were present in BT culture supernatant. The suppressive effect was retained after heating at 100 °C for 10 min (Figure 2a). Size fractionation using Amicon^®^ spin column revealed that the sizes of inhibitors were predicted to be 10–100 kDa (Figure 2b).

### 2.3. Construction of a Tn4351-Based Mutant Library and Screening to Identify the BT Genes Encoding Anti-Toxigenic Factors Against CD

We constructed a BT mutant library by employing Tn*4351* to identify those BT genes attributable to CD anti-toxigenicity. A total of 1392 clones were screened with a Vero cell cytotoxicity assay. We identified twenty BT mutants where the inhibitory effect was reduced to less than 40% of the parent strain (Figure S2). These mutants were screened again to confirm the results, and 15 clones were confirmed to reduce the anti-toxigenicity to less than 40% of the BT parent strain (Table S1). Of these, five had insertions associated with polysaccharide biosynthesis (genes in capsular polysaccharide biosynthesis loci of PS-4 and PS-6), degradation (α-galactosidase gene), and methylerythritol 4-phosphate pathway (MEP) for isoprenoid biosynthesis (*gcpE or ispG*). Since MEP is involved in the biosynthesis of undecaprenyl phosphate, a lipid carrier that transports glycan across the membrane, we focused on *gcpE*, which encodes 1-hydroxy-2-methyl-(E)-butenyl 4-diphosphate synthase. The *gcpE* mutant of BT (designated as Δ*gcpE*) was used in the following experiments.

### 2.4. Characterization of gcpE Mutant of BT

**Figure 3:**
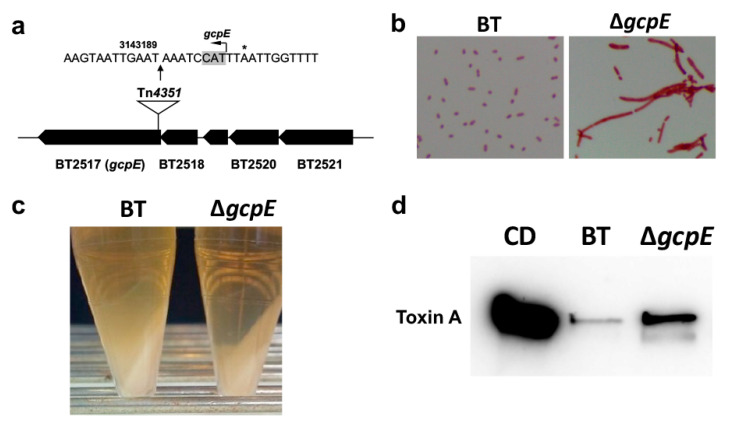
Characterization of the Δ*gcpE* mutant strain. (**a**) The Tn*4351* insertion site in the Δ*gcpE* mutant. Tn*4351* was inserted into the 5′ end of *gcpE*. * Stop codon of the upstream gene (BT2518). (**b**) Gram stain of wild type BT and Δ*gcpE* strains. (**c**) Mucoid layer in BT culture following centrifugation of either BT WT or Δ*gcpE*. (**d**) Toxin A levels in CD culture alone, exposed to the supernatant of wildtype BT or Δ*gcpE*.

Tn*4351* was inserted into the 5′ region of *gcpE* (BT2517) in the Δ*gcpE* mutant strain (Figure 3a). The Δ*gcpE* growth rate was not different from the parent strain (data not shown). However, the cells were elongated, probably due to an impairment in cell wall septum formation (Figure 3b). In addition, the mucous layer that could be observed in the parent strain after centrifugation was decreased in the Δ*gcpE* mutant strain (Figure 3c). These findings indicate that transmembrane glycan transport in the Δ*gcpE* strain was negatively affected. The inhibitory effect on CD toxin production, which was observed in the parent BT strains, was also reduced in the Δ*gcpE* strain (Figure 3d). These data indicate that either cell wall glycans or exopolysaccharides, whose transmembrane transport is dependent on undecaprenyl pyrophosphate, are associated with the inhibitory effect of CD toxin production.

### 2.5. Inhibitory Effect of the Polysaccharide Fraction from BT Culture on CD Toxin Production

**Figure 4:**
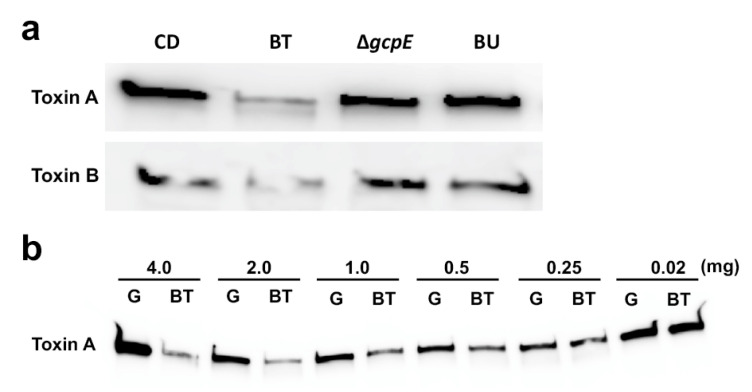
Inhibitory effect of the BT culture supernatant polysaccharide fraction (PF). (**a**) PFs from the indicated strains were used to prepare conditioned medium. After CD was cultured in GAM (indicated by CD) or conditioned medium containing 2.0 mg/mL of PF from each strain, toxin A and B levels in the CD culture supernatants were evaluated by Western blotting. BT, *B. thetaiotaomicron* VPI-5482; Δ*gcpE*, Tn*4351*-inserted *gcpE* mutant; BU, *B. unifromis* ATCC9492. (**b**) Dose-dependent inhibition of BT-derived PF on CD toxin production. PFs from GAM (indicated by G) or BT was added to the medium at the indicated concentration. After CD was cultured in the conditioned media for 24 h, the amount of toxin A in the CD culture supernatants was evaluated by Western blotting.

We prepared the polysaccharide fraction (PF) from GAM broth and the culture supernatants of BT, Δ*gcpE*, and *B. uniformis* ATCC8492 (BU). BU was used as a negative control since the species had no effect on CD toxin production (Figure 1c,d). The culture supernatants of these strains were dialyzed in membrane with a molecular weight cut-off of 3.5 K, followed by proteinase K treatment. After the material was lyophilized, the inhibitory effect on CD toxin production was compared across the samples. BT VPI-5482-derived PF (2.0 mg/mL) suppressed CD toxin production while PFs from the Δ*gcpE* and BU strains showed no inhibitory effect (Figure 4a). The BT-derived PF inhibitory effect was observed at concentrations above 0.5 mg/mL (Figure 4b). These data indicate that BT-derived polysaccharides are involved in the suppression of CD toxin production.

### 2.6. Transcriptional Analysis of PaLoc in CD

**Figure 5:**
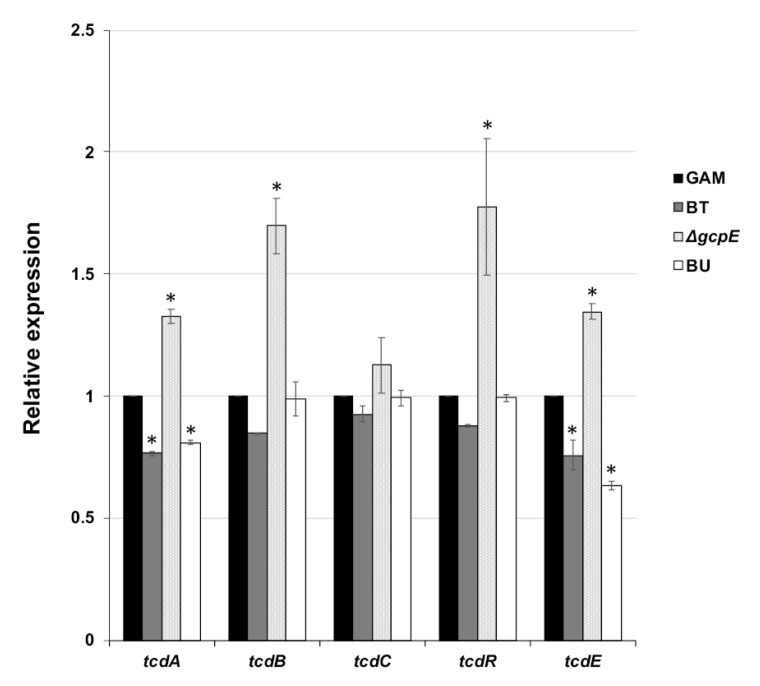
Effect of BT-derived PFs on PaLoc gene expression. After CD was cultured to late-logarithmic phase in the conditioned medium containing 2.0 mg/mL PFs from the indicated strains, the RNA was extracted from each sample. RT-qPCR was performed to examine the change in gene expression for toxins A and B (*tcdA* and *tcdB*), the positive- and negative-regulators (*tcdR* and *tcdC*), and the holin-like protein (*tcdE*). The relative expression of each gene to the control (expression in the conditioned medium prepared with PF from GAM broth) is shown. Significant difference compared with GAM is indicated by * (*p* < 0.01).

We determined the transcriptional alteration of the CD PaLoc genes in response to PFs prepared from GAM broth and the culture supernatant of the BT, Δ*gcpE*, and BU strains. CD was cultured in the PF-conditioned media for 9 h prior to RNA extraction, and qPCR for each gene transcript was performed. The expression of *tcdA* and *tcdE*, which encode toxin A and a holin-like protein, respectively, was slightly decreased in the presence of BT- and BU-derived PFs compared to the CD control (Figure 5). In the presence of Δ*gcpE*-derived PF, the PaLoc gene expression levels, excepting *tcdC*, were slightly elevated. However, the overall effect of these PFs on the PaLoc gene expression levels was limited.

### 2.7. CD Toxin Localization Following Exposure to BT-Derived PF

**Figure 6:**
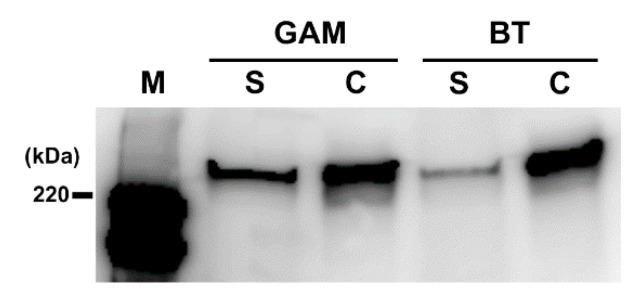
Localization of Toxin A in CD culture. After CD was cultured in the conditioned medium containing PF (2.0 mg/mL) derived from GAM broth or BT, the culture was separated into the supernatant and cell pellet by centrifugation. Toxin A levels in the supernatant (S) and cell pellet (P) were evaluated by Western blotting with anti-Toxin A antibody. M, HRP-labeled protein size marker.

Due to the limited effect of BT-derived PF on PaLoc gene expression, we examined the localization of CD toxins following incubation in the conditioned media. These results indicated that CD toxins accumulated inside the cell but decreased in the culture supernatant when exposed to BT-derived PF (Figure 6). We stained CD cultured in the conditioned media and compared microscopic images to assess levels of CD cell autolysis. The CD cells stained as Gram negative at 12 h post-incubation due to autolysis. CD continued to stain Gram positive following co-cultivation with BF and BT (Figure S3), indicating that BT suppresses CD toxin release by inhibiting cell autolysis.

### 2.8. Autolysis Assay

**Figure 7:**
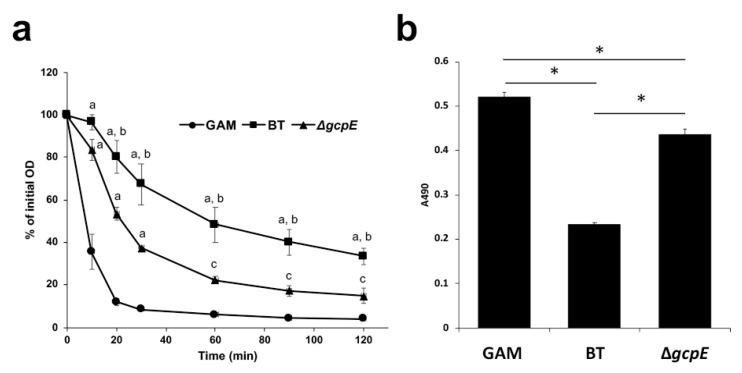
**Autolysis assay of CD.** (**a**) CD strain 630 was cultured in GAM broth (closed circle) or the conditioned medium prepared with culture supernatants derived from BT (closed square) or Δ*gcpE* (closed triangle). The CD cells were suspended in PBS containing 0.02% Triton X-100. Cell lysis was monitored by a decrease in OD_590_. The percent reduction in the initial OD_590_ was calculated every 10 or 30 min until 2 h post-incubation. At each time point, significant differences with *p*-value less than (a) 0.01, and (c) 0.05 when compared with the control (CD cultured in GAM); (b) significant differences between BT and Δ*gcpE* with a *p*-value less than 0.01. (**b**) Lactate dehydrogenase (LDH) activity released into the 24-h culture supernatant of CD in GAM or the conditioned medium prepared with culture supernatant of BT or Δ*gcpE*. LDH activity was determined using CytoTox 96 Non-Radioactive Cytotoxicity Assay kit (Promega), and the signal from the test was recorded as absorbance at 490 nm. * Significant difference with *p*-value less than 0.01.

To confirm our hypothesis that BT suppresses CD cell autolysis, an autolysis assay using Triton X-100 was performed (Figure 7a). In this assay, we used the CD strain 630 since this strain undergoes autolysis more easily than CD ATCC9689. After CD cells were cultured in conditioned media prepared with BT culture supernatant (wild type or Δ*gcpE*), the cells were collected by centrifugation and suspended in phosphate-buffered saline containing 0.02% Triton X-100. The decline in OD_590_ was monitored periodically. CD alone was rapidly lysed by the detergent. Conversely, the CD cells cultured in conditioned media containing BT culture supernatant were significantly more resistant to the detergent. Supplementation with Δ*gcpE* culture supernatant partially reduced CD resistance to the detergent. After CD ATCC9689 was cultured in GAM or conditioned medium prepared with BT culture supernatant (wild type or Δ*gcpE*) for 24 h, lactate dehydrogenase (LDH) activity released into the CD culture supernatant was measured as a cytoplasmic marker (Figure 7b). Consistent with the findings of autolysis assay, LDH activity in the CD culture supernatant was significantly lower in the medium containing BT culture supernatant (wild type or Δ*gcpE*) than that in GAM broth. However, the LDH activity released from CD was much lower in the conditioned medium containing wild type BT culture supernatant than in that containing Δ*gcpE* culture supernatant.

### 2.9. Effect of Lysozyme Treatment

**Figure 8:**
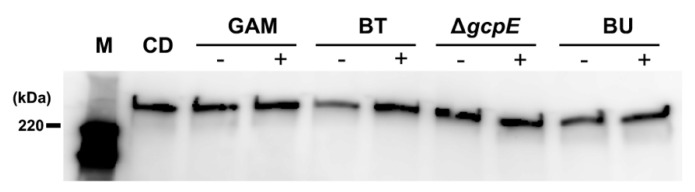
**Lysozyme treatment reduced the suppressive effect of BT-derived PF on CD toxin production.** PFs from either GAM broth or the culture supernatants of *B. thetaiotaomicron* VPI-5482 (BT), Tn*4351*-inserted *gcpE* mutant of BT (Δ*gcpE*), or *B. uniformis* ATCC8492 (BU) were treated with lysozyme. CD Toxin A production in the conditioned media, which contained PFs with or without lysozyme treatment (indicated by + and -, respectively), was compared. CD, CD culture supernatant in GAM broth; M, HRP-labeled protein size marker.

To determine the role of BT cell wall-associated glycan on CD toxin production, PF was treated by lysozyme. The inhibitory effect of BT-derived PF was ameliorated by lysozyme treatment (Figure 8). However, the inhibitory effect of BT-derived PF was not affected by polymyxin B treatment (data not shown), indicating that cell-wall associated glycan is involved in the suppressive effect of BT on CD toxin production.

## 3. Discussion

CDAD is a leading cause of nosocomial diarrhea that can cause life-threatening colitis in elderly or immunocompromised patients. CDAD has been recognized as one of the most alarming nosocomial infections due to its severe diarrhea and high transmissibility to other patients. Although antibiotic treatment disrupts the gut microbiota, making it the greatest risk factor for CDAD, vancomycin or metronidazole are the first line drugs for CDAD. Such antibiotic treatment is effective for CDAD, but nearly 20% of cases relapse and the recurrent cases are less responsive to vancomycin and metronidazole treatments [22].

The successful clinical effects of fecal microbiota transplantation on recurrent CDAD have attracted the attention of scientists to the mechanism of colonization resistance to CD by the human gut microbiota [30]. In this study, we found that a human gut microbe, BT, inhibits CD toxin production in vitro. This effect was observed even with cell-free culture supernatant. A preliminary analysis indicated that heat-stable molecules with a relatively large molecular size (10–100 kDa) present in BT culture supernatant were responsible for the suppression of CD toxin production (Figure 2). The culture supernatants from clinical isolates of *B. thetaiotaomicton* also showed a suppressive effect on CD toxin production (Figure S1), indicating that the inhibitory molecules are conserved within the species. Interestingly, *B. fragilis* (BF) also suppressed CD toxin production, but the effect was weaker than BT. On the other hand, BV and BU showed no effect. These results correlated well with the taxonomic relatedness among the species as BF is phylogenetically closer to BT than BV or BU (Figure S4). These data indicate that similar inhibitory molecules were conserved in BT and its closely related species. Deng et al. reported that BF suppressed gut inflammation evoked by CD through a host-oriented effect that improves gut barrier function [29]. The authors concluded that capsular polysaccharide, PSA, is responsible for the suppression of CD pathogenicity. It is possible that the PSA or cell wall-associated glycans of BF may suppress CD autolysis as described here, which may partly contribute to prevent CD infection.

We performed transposon mutagenesis in BT using Tn*4351* to identify the genes responsible for anti-CD toxin production. Despite the small sample size screened (1392 clones screened), we identified candidate clones that reduced the suppressive effect on CD toxin production. This high detection rate might be due to the involvement of multiple genes or operons required to synthesize the effective molecules. In fact, several clones identified had transposon insertions in genes associated with capsular polysaccharide biosynthesis (PS-4 and PS-6) and the MEP pathway, 23, 20, and 7 gene products, respectively. In addition, the genes for the cell-division protein that is involved in peptidoglycan remodeling or β-galactosidase, were also included in the list of the candidate genes (Table S1), indicating that BT glycan metabolism is involved in the suppressive effect on CD toxin production.

Among the genes identified by transposon mutagenesis, we focused on *gcpE* (BT2517), which encodes an enzyme of the MEP pathway. The MEP pathway supplies the isoprenoid backbone to synthesize the undecaprenyl phosphate lipid carrier that is crucial in the transmembrane transport of cell surface glycans. Despite repeated trials to make a null mutant of *gcpE* by homologous recombination, we failed to obtain a mutant. However, *gcpE* could be deleted from the chromosome during plasmid complementation of the gene (data not shown). Therefore, we concluded that *gcpE* is an essential gene for BT. Since Tn*4351* was inserted into the 5′ end of *gcpE* (Figure 2a), we predicted synthesis of an N-terminally truncated GcpE protein. However, a malfunction in glycan transport was apparent from the cell elongation (impaired septum formation) and the reduced production of exopolysaccharides (Figure 2b,c). The transposon insertion in *gcpE* partially abrogated the suppressive effect of BT on CD toxin production (Figure 2d). Loss of the suppressive effect was also observed with the deproteinized, lyophilized PF from the Δ*gcpE* culture supernatant, while the same preparation from the parent strain retained its suppressive effect. Lysozyme, but not polymyxin B, treatment abrogated the suppressive effect of the BT-derived PF. Based on these results, we concluded that BT extracellular glycans, especially cell wall-associated glycans, suppressed CD toxin production.

It has been reported that PaLoc gene expression is repressed by carbon catabolite repression protein (CcpA) in the presence of rapidly metabolizable sugars such as glucose [31]. However, catabolite repression is unlikely to be involved in the suppressive effect of BT since expression levels of PaLoc genes showed only limited differences in expression with or without BT-derived PF (Figure 5). In addition, the PF was dialyzed using a membrane with a MW cut-off of 3.5 K, indicating that neither simple sugars nor oligosaccharides are related to the suppressive effect. The expression of genes on the PaLoc locus, except for the negative regulator *tcdC*, was slightly increased during CD growth in the conditioned medium with Δ*gcpE*-derived PF when compared to the control (GAM-broth-derived PF). This might be due to an alteration in the carbohydrate pattern on the cell wall caused by malfunctional glycan transport in the Δ*gcpE*.

Consistent with findings that BT did not affect PaLoc gene expression (Figure 5), CD toxin levels were decreased in the culture supernatant but not inside the cells (Figure 6). Thus, we considered the possibility that CD toxin undergoes autoprocessing in the presence of BT cell-wall-derived glycans. Both CD toxins A and B possess a cysteine protease domain [32,33] that is activated when it encounters inositol hexakisphosphate (myo-inositol) in the host membrane, which releases the glycosyltransferase domain into the host cell cytoplasm [32,34]. However, this hypothesis was not supported by the Western blotting analysis, in which only a single band of unprocessed toxin was obtained but no proteolytic product was detected.

Finally, we hypothesized that BT cell wall-associated glycans inhibit the cell autolysis of CD. Gram staining and autolysis assays supported this hypothesis, in which BT inhibited CD autolysis (Figure 7 and Figure S3). CD toxins do not have a conventional secretion signal peptide, and the secretion machinery for CD toxins has not been identified on the CD genome [35]. Therefore, we considered that CD cell autolysis is involved in the extracellular release of toxins A and B. Although the holin-like protein encoded for by *tcdE* has been reported to be necessary for CD toxin release in TY medium [14,36], Olling et al. have reported that TcdE is not necessary for toxin secretion [37]. Recently, it was demonstrated that a novel peptidoglycan-degrading enzyme, Cwp19, is involved in CD toxin release through stationary-phase autolysis [38]. CD toxin release was independent on TcdE when CD was cultured in rich medium such as brain-heart-infusion broth (BHI), and CD toxins were extracellularly released by stationary-phase autolysis in BHI [37,38]. These findings indicate that CD releases toxins through two different ways depending on nutritional availability. Since we cultured CD in rich medium (GAM broth), CD toxin release might depend on stationary-phase autolysis, which mediated by peptidoglycan hydrolases such as Cwp19. Many uncharacterized cell wall hydrolase genes are present on the genomes of Gram-positive bacteria. Since BT showed a limited effect on *tcdE* expression, BT cell wall-associated glycans might inhibit stationary-phase CD autolysis. Cwp19 preferentially cleaved acetylated *N*-acetylglucosamine [38]. Difference in the effect on CD cell autolysis among *Bacteroides* species might be due in part to the diversity in cell surface glycan modification.

Due to the clinical importance of CD, many studies have been performed to elucidate the mechanisms of gut microbiota that antagonize the pathogenicity of CD. It has been demonstrated that probiotic bacteria such as *Bifidobacterium* and *Lactobacillus* inhibit CD virulence by affecting the growth, germination, sporulation, or toxin gene expression [39,40,41]. In addition, Castagliuolo et al. reported that the 54-kDa protease of *Saccharomyces bourardii* degrades both CD toxin and its brush border membrane receptor, thus protecting rats from CD enteritis [42]. However, the clinical effect of probiotics on CDAD is controversial [43,44]. Further efforts to explore the detailed mechanisms by which human gut microbes antagonize CD are necessary to establish strategies to control refractory CDAD.

In summary, we have herein reported that BT cell wall-associated glycans repress CD toxin release by inhibiting cell autolysis. Bacterial cell autolysis releases bacterial cellular components (e.g., flagella and peptide glycan fragments) as well as intracellular molecules (e.g., DNA and enzymes). These pathogen-associated molecular patterns are recognized by host Toll-like receptors and evoke inflammation [45]. *Bacteroides* species produce multiple phase-variable extracellular polysaccharides that decorate its cell surface [46]. These phase-variable polysaccharides are considered to play a role in evading host immunity. In this study, we report the novel function of BT glycans to reduce CD virulence. Bacterial exopolysaccharides and peptide glycans play a role in cell-to-cell communication that influences physiological processes such as biofilm formation and spore germination [47,48]. BT may utilize surface glycans to both evade host immunity and communicate with diverse members of the gut microbiota to maintain gut homeostasis. Further study is required to identify the molecular structure of the BT cell wall-associated glycan(s) reported here, and the alteration of CD transcriptome in response to the glycan(s) is helpful to design novel prophylaxes or therapeutics for CDAD, one of the important complications of antibiotic treatment.

## 4. Materials and Methods

### 4.1. Bacterial Strains and Growth Conditions

*Clostridium difficile* (CD) toxigenic strains ATCC9689 and 630 [35] were used; both strains produce toxins A and B. The *Bacteroides* species used were *B. thetaiotaomicron* (strain VPI-5482 and seven clinical isolates), *B. fragilis* (NCTC9343 and YCH46 [49]), *B. ovatus* ATCC8483, *B. uniformis* ATCC8492, and *B. vulgatus* ATCC8482. *Bifidobacterium longum* JCM1217, *Clostridium ramosum* JCM1298, and *Escherichia coli* strain DH5α were also tested as other representative intestinal bacteria. All bacterial strains used were grown anaerobically in GAM (Gifu anaerobic medium, Nissui Pharmaceutical Co., Tokyo, Japan) broth or on GAM agar plates at 37 °C. Anaerobic cultivation was performed using an anaerobic chamber (Forma Scientific model 1025) conditioned with mixed gas (CO_2_, 80%; N_2_, 10%; H_2_, 10%).

### 4.2. Preparation of Conditioned Medium

The filtrate of culture supernatant of the selected bacteria was prepared to test the effect on CD toxin production. A single colony of each bacterial strain grown on GAM agar plates was inoculated into 10 mL GAM broth, and the media were anaerobically cultivated overnight at 37 °C. A part of the overnight culture (0.1 mL) was inoculated into 10 mL of freshly prepared GAM broth and incubated again for 24 h. The culture was then centrifuged at 10,000× *g* for 15 min, and the supernatant was passed through a 0.22 µm filter (ADVANTEC, DISMIC-25SS) to remove bacterial cells. Aliquots of the filtrate were stocked at −80 °C until use. Conditioned medium was prepared by mixing the filter sterilized culture supernatant with an equal volume of fresh GAM broth. Another type of conditioned medium that contained the polysaccharide fraction from the selected bacterial culture was prepared as described below (Section 4.10).

### 4.3. Cultivation of CD in Modified Medium

Frozen stock (−80 °C) of the CD was streaked onto GAM agar plates and anaerobically cultured for 48 h at 37 °C. A single colony of each CD strain was precultured overnight in GAM broth, and 0.8 mL of the culture was inoculated into 10 mL each of conditioned media. The media were anaerobically incubated at 37 °C until the optical density at 600 nm reached 2.0, the culture supernatant was collected by centrifugation at 10,000× *g* for 15 min and then passed through a 0.22 µm filter to remove CD cells. The supernatants and cell pellets were stored at −80 °C until CD toxin levels were determined by cytotoxicity assay and Western blotting.

### 4.4. Microscopy Observation

Equal volumes (0.1 mL each) of stationary-phase CD ATCC9689 and *B. fragilis* YCH46 or *B. thetaiotaomicron* VPI-5482 were inoculated into 10 mL fresh GAM broth. We monitored the visible changes of CD cells under co-cultivation with *B. fragilis* YCH46 or *B. thetaiotaomicron* VPI-5482. The co-culture was periodically sampled and spread on glass slides. After fixation with methanol, the slides were Gram stained using a staining kit (Favor G “Nissui”; Nissui Pharmaceutical Co., Ltd., Tokyo, Japan). Microscopic images were captured with a Leica ICC50 HD microscope. After 24-h incubation, the remaining cultures were centrifuged at 10,000× *g* for 15 min, and the supernatant was passed through a 0.22 µm filter (ADVANTEC, DISMIC-25SS) to remove bacterial cells. Aliquots of the filtrate were stocked at –80 °C until use for cytotoxicity assay described below.

### 4.5. Cytotoxicity Assay

The CD toxins produced in GAM or conditioned medium were detected by a cytotoxicity assay as described previously [50]. Cytopathic effect by Toxin A and B was examined using HT-29 and Vero cells, respectively. HT-29 (2.0 × 10^4^ cells) and Vero cells (1.0 × 10^4^ cells) were seeded in 24-well plates containing 1 mL of Dulbecco’s Modified Eagle’s Medium (DMEM; Sigma-Aldrich Japan, Tokyo, Japan) supplemented with 10% (*v*/*v*) heat-inactivated fetal bovine serum (FBS; Sigma-Aldrich Japan, Tokyo, Japan), 100 U/mL penicillin, and 100 μg/mL streptomycin at 37 °C for 24 h under 5% CO_2_. The media was then replaced with 1 mL of DMEM containing 10% FBS, 100 U/mL penicillin, 100 μg/mL streptomycin, and 50% (*v*/*v*) of either the selected bacterial culture supernatant or GAM broth as a control. After the cells were incubated at 37 °C for 24 h under 5.0% CO_2_, the cytopathic effect was evaluated by morphological change. Intact cell population was also estimated by neutral red staining. Neutral red was purchased from Wako Chemical Co. Ltd., Tokyo, Japan. In brief, after the cells were washed with saline, 1 mL of fresh media containing neutral red (final concentration of 0.05%) was added to each well and incubated for 1 h at 37 °C under 5.0% CO_2_. The media were carefully removed, and the cells were washed three times with saline. The neutral red incorporated into the cells was then eluted with 0.5 mL of solution containing 50% (*v*/*v*) ethanol and 1% acetic acid. The multi-well plates were shaken for 1 min and the absorbance at 540 nm was read in a microliter plate reader (SH-9000, Corona Electric, Ibaraki, Japan).

### 4.6. Detection of Toxins A and B by Western Blotting

The CD culture supernatants and cell pellets in the conditioned media were subjected to SDS-PAGE, and the proteins were transferred to PVDF membranes (Hybond-P; GE Healthcare). The membranes were incubated overnight in PBS-T (PBS containing 0.1% Tween 20) containing 3% dry milk powder and then incubated with affinity-purified monoclonal mouse antibodies specific for *C. difficile* toxin A (Abcam) or affinity-purified polyclonal rabbit antibodies specific for *C. difficile* toxin B (Abnova). Toxin-bound antibodies were then detected with peroxidase-conjugated secondary antibodies (BIO-RAD) and the ECL prime Western Blotting Detection Reagent (GE Healthcare), according to the manufacturer’s instructions.

### 4.7. Characterization of the Inhibitory Components in BT Culture on CD Toxigenicity

The filter-sterilized overnight cultures of either *B. thetaiotaomicron* VPI-5482 or *E. coli* DH5α were heated at 100 °C for 10 min to test the heat stability of the target components. Size fractionation of the filter-sterilized BT culture using Amicon^®^ Ultra Centrifugal Filter Units (Millipore) with a molecular weight (MW) cut-off of 3 K, 10 K, or 100 K was then performed to estimate the approximate molecular sizes of the target components. These samples were used to prepare the conditioned media to culture CD. CD toxin levels in each of the conditioned media were evaluated by Western blotting as described above.

### 4.8. Transposon Mutagenesis

The transposon mutant library of *B. thetaiotaomicron* VPI-5482 was constructed with Tn*4351* as described previously [51]. The Tn*4351* delivery plasmid pEP4351 was introduced into *B. thetaiotaomicron* VPI-5482 by electroporation. Electrocompetent cells were prepared from the culture at stationary phase (48-h culture). Competent cells were pulsed using a Gene-Pulser II apparatus (Bio-Rad) with the following parameters: 12.5 kV/cm, 200 Ω, and 25 μF. The transposon-insertion mutants were selected on GAM agar plates supplemented with 10 μg/mL erythromycin. Individual colonies were seeded into 96-deep-well plates and grown anaerobically overnight in GAM broth containing erythromycin (10 μg/mL). A part of the culture was frozen in 25% glycerol and stored at −80 °C until use. The deep-well plates were then centrifuged (6000× *g* for 15 min at 4 °C), and the supernatants were used to prepare the conditioned media for CD cultivation. CD toxin levels in each of the cultures were evaluated by cytotoxicity assays using HT29 cells as described above.

### 4.9. Identification of Transposon Insertion Site

The transposon insertion sites in the Tn*4351*-based mutant library of *B. thetaiotaomicron* VPI-5482 were identified by nested arbitrarily primed PCR (AP-PCR), as described previously [52]. Overnight cultures of the mutants were mixed with 20 μL of 50 mM NaOH and boiled for 10 min. The samples were then neutralized with an equal volume of 80 mM Tris-HCl (pH 7.0) and centrifuged (6000× *g* for 10 min at 4 °C). The supernatants (1 μL) were used as templates for AP-PCR. The first round PCR was performed with a random primer (AR7 or AR8) and a transposon specific primer, S3794 (Table S2). To increase the specificity, the second round of PCR was done using the AR2 primer, which has an identical sequence to the 3′end of AR7 or AR8, and ISF (a transposon-specific primer designed outside S3794). The AP-PCR workflow performed in this study is shown in Figure S5. The product of the second round of PCR was then purified and sequenced from the right arm of the transposon into the chromosomal DNA using the sequence primer IS4908-S. A BLASTN search was performed with the obtained sequences against the *B. thetaiotaomicron* VPI-5482 genome (GenBank accession numbers NC_004663). Only the mutants from which AP-PCR-obtained sequences aligned with the *B. thetaiotaomicron* genome and Tn*4351* were included in the analysis. DNA sequencing was performed on an ABI Prism 3730 Genetic Analyzer (Applied Biosystems) using the ABI Prism BigDye Terminator Cycle Sequencing Ready Reaction Kit (version 1.1; Applied Biosystems).

### 4.10. Partial Purification of Polysaccharide Fraction from Culture Supernatant

The culture supernatant of selected *Bacteroides* strains in GAM broth was packed into dialysis membranes (MECO 3500, BioDesign, NY, USA) and dialyzed against deionized water for 72 h at 4 °C. The dialyzed samples were treated with 10 µg/mL of proteinase K (Takara Shuzo Co., Ltd., Otsu, Japan) for 24 h at 25 °C. After additional dialysis under the same conditions for 72 h, the samples were lyophilized using FDU-2200 (−80 °C, 10 Pa, Tokyo Rikakikai, Tokyo, Japan). Each lyophilized sample was reconstituted by dissolving 2 mg/mL into GAM broth, passed through a 0.22 μm filter, and used to prepare conditioned media. Filter sterilized samples were heated at 95 °C for 10 min to inactivate proteinase K. The purified polysaccharide fraction was treated by 0.1 mg/mL lysozyme at 37 °C for 6 h when necessary. After the inactivation of lysozyme (Sigma-Aldrich Japan, Tokyo, Japan) at 95 °C for 30 min, the sample was centrifuged (20,000× *g*, 5 min, 4 °C). The supernatant was used to prepare conditioned media.

### 4.11. RNA Extraction and Reverse Transcription Quantitative PCR (qPCR)

Total RNA was extracted using the RNeasy MinElute Cleanup kit (QIAGEN). RNA concentration was determined using a NanoDrop Spectrophotometer (Thermo Scientific) after a 50-fold dilution in RNase-free water. Following the adjustment of the total RNA concentration to 400 ng/µL, 2 µL of each RNA sample was reverse transcribed with PrimeScript^®^ RT reagent kit (Takara Shuzo Co., Ltd., Otsu, Japan) with random hexamers in a final volume of 20 µL at 37 °C for 15 min. The reaction was stopped by heating the sample at 85 °C for 5 sec. Three qPCRs were carried out on diluted cDNA from each sample using the ABI StepOne Plus real-time PCR system (Applied Biosystems) in microcapillary tubes in a final volume of 20 µL. The forward and reverse primers used for amplification of the fragments of *tcd A*, *tcd B*, *tcd C*, *tcd R,* or *tcd E* are listed in Table S2.

### 4.12. Autolysis Assay

To estimate the abundance of CD cells entering the lytic cycle, the autolysis assay was performed as described by Camiade et al. [53]. Then, CD 630 was cultured in the conditioned media prepared with the filter-sterilized culture supernatant of *B. thetaiotaomicron* VPI-5482 (wild type or Δ*gcpE*) until it reached the exponential growth phase (OD_590_ = 1.0). A part of the culture (0.8 mL) was collected into 1.5 mL tubes and centrifuged (10,000× *g*, 2 min, 4 °C). After the cell pellet was washed twice with PBS (pH 7.4), it was suspended in 1 mL of PBS containing 0.05% Triton X-100 (Sigma-Aldrich Japan, Tokyo, Japan). The suspension was incubated at 37 °C in an anaerobic chamber. The reduction in OD_590_ of the suspension was monitored periodically (0 min, 10 min, 20 min, 30 min, 60 min, 90 min, and 120 min) post-suspension.

### 4.13. Measurement of Lactate Dehydrogenase (LDH) Activity

To assess the CD cell autolysis, LDH activity in the culture supernatant was measured as a cytoplasmic marker. CD ATCC9689 was cultured in the conditioned media prepared with the filter sterilized culture supernatant of *B. thetaiotaomicron* VPI-5492 (wild type or Δ*gcpE*). After 24-h cultivation, the culture supernatant was collected by centrifugation (10,000× *g*, 2 min, 4 °C). LDH activity in the culture supernatant was determined using a CytoTox 96 Non-Radioactive Cytotoxicity Assay kit (Promega) according to the recommendations of the supplier.

### 4.14. Statistical Analysis

Data were expressed as the mean ± standard deviation from three independent repeats. Statistical analysis of the data was performed with StatFlex ver. 6.0 (Artech Co., Ltd., Tokyo, Japan) using an analysis of variance (ANOVA) followed by a Dunnett’s or Tukey’s test. Data were considered to be significantly different if the *p* value was less than 0.05.

## Data Availability

Data is contained within the article.

## Supplementary Materials

The following are available online at https://www.mdpi.com/2079-6382/10/2/187/s1, Table S1: Tn*4351*-insertion sites into the BT chromosome where anti-toxigenicity against CD was reduced to less than 40% of the parent strain, Table S2: PCR primers used in this study. Figure S1: The suppressive effect of BT culture supernatants on CD toxin A production. Figure S2: Distribution of the relative inhibitory effect on CD toxin production by Tn*4351*-inserted mutants compared to the parent strain (BT VPI-5482). Figure S3: Temporal changes in the Gram stain retention of CD cell walls. Figure S4: The phylogenetic relationship of *Bacteroides* species used in this study. Figure S5: Outline of the AP-PCR and nucleotide sequencing workflow used to identify Tn*4351*-insertion sites in the BT VPI-5482 genome.
