# Supplementary material for: The Human Gut Microbe Bacteroides thetaiotaomicron Suppresses Toxin Release from Clostridium difficile by Inhibiting Autolysis
Source: Antibiotics (Basel). 2021 Feb 15;10(2):187. doi: 10.3390/antibiotics10020187 (PMC7918992; doi:10.3390/antibiotics10020187)
Supplement: Supplementary file 1 [file antibiotics-10-00187-s001.zip › Supplemental Materials.pdf]

Supplemental Materials

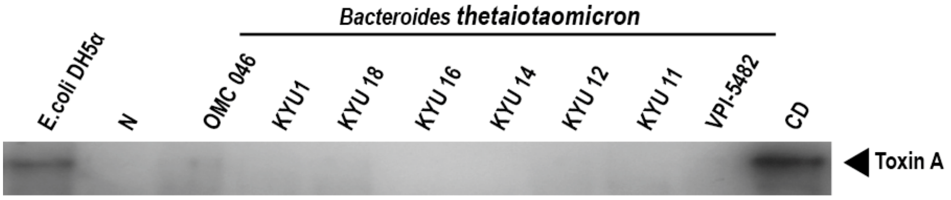

Figure S1. The suppressive effect of BT culture supernatants on CD toxin A production. The conditioned media was prepared by mixing equal parts fresh GAM broth and the culture supernatant of the indicated strain, excepting CD, which was cultured in GAM broth alone. After CD was cultured in each medium, the cell-free supernatant was used for Western blotting with an anti-Toxin A antibody. N, medium control (GAM broth alone).

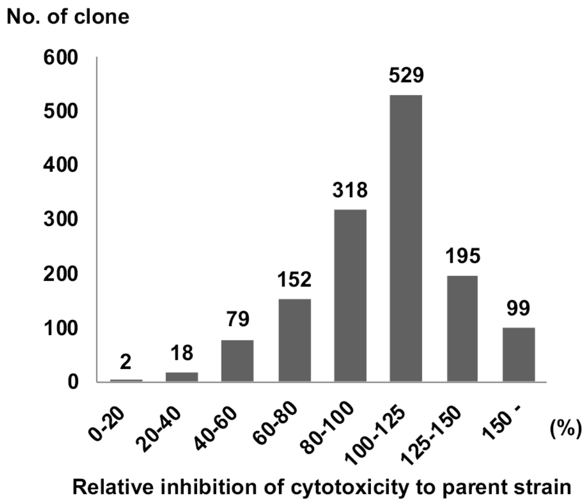

Figure S2. Distribution of the relative inhibitory effect on CD toxin production by Tn*4351*-inserted mutants compared to the parent strain (BT VPI-5482). The relative cytotoxicity of each mutant was calculated from a neutral red assay, in which 1,392 mutants were examined. The number of clones included in the analysis (y-axis) and the indicated range of relative inhibition (x-axis) are shown.

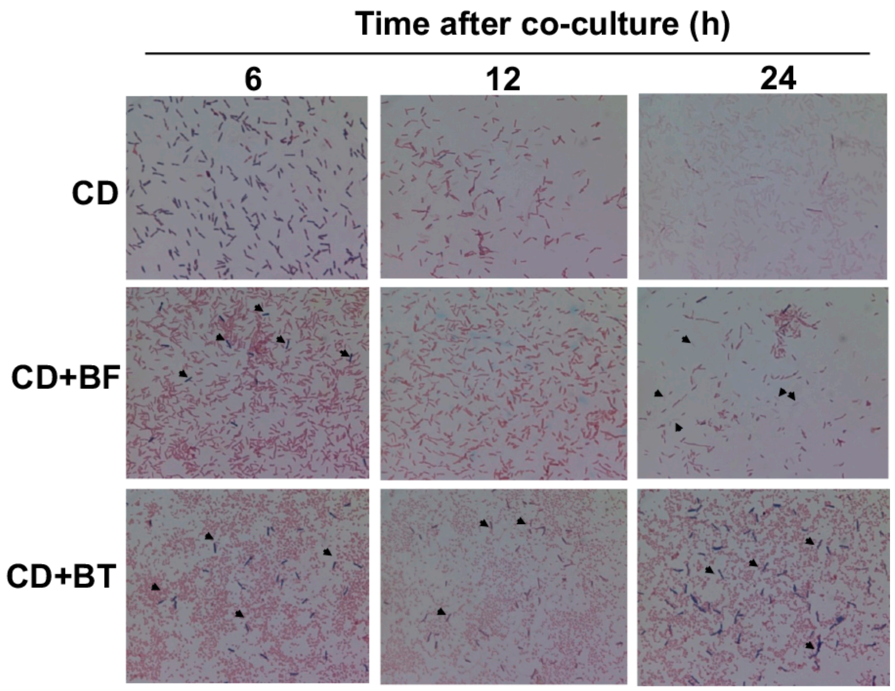

Figure S3. Temporal changes in the Gram stain retention of CD cell walls. A Gram stain of CD cells cultured in GAM (upper panels) or co-cultured with BF (middle panels) became faint as cultivation time progressed. CD cells co-cultured with BT (bottom panels) stained Gram positive even after 24 h of incubation. Arrowheads indicate CD cells in the co-culture with the respective *Bacteroides* species.

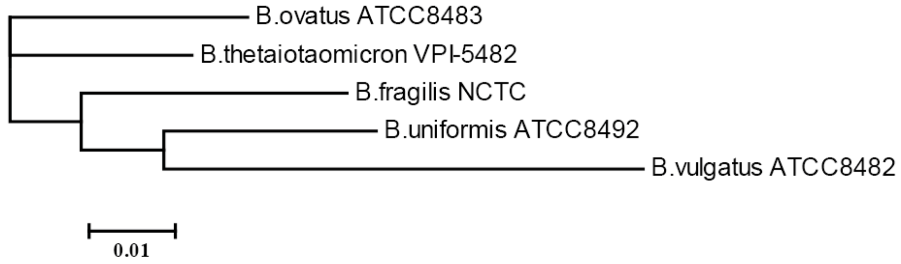

Figure S4. The phylogenetic relationship of *Bacteroides* species used in this study. The 16S ribosomal RNA sequences of the five selected *Bacteroides* species were downloaded from RDP Release 11 (http://rdp.cme.msu.edu) and aligned with ClustalW (https://clustalw.ddbj.nig.ac.jp). The phylogenetic tree was drawn with MEGA6 software (Molecular evolutionary genetics analysis version 6.0) based on the distance matrix calculated by ClustalW. Accession numbers in the NCBI database of the 16S ribosomal RNA gene sequences for *B. fragilis* NCTC9343, *B. ovatus* ATCC8483, *B. thetaiotaomicron* VPI-5482, *B. uniformis* ATCC8492 and *B. vulgatus* ATCC8482 are CR626927, AB050108, AE015928, AB050110 and CP000139, respectively. Scale bar indicates the genetic distance.

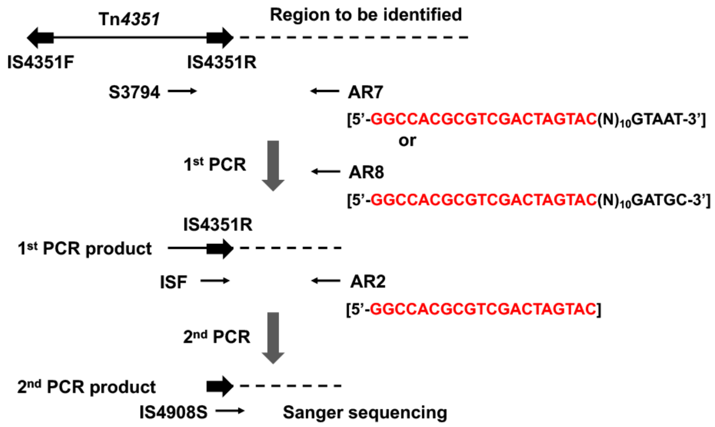

Figure S5. Outline of the AP-PCR and nucleotide sequencing workflow used to identify Tn*4351*-insertion sites in the BT VPI-5482 genome. Identical nucleotide sequences are shown in red.
